# Green phosphorescent organic light-emitting diode exhibiting highest external quantum efficiency with ultra-thin undoped emission layer

**DOI:** 10.1038/s41598-021-86333-9

**Published:** 2021-04-19

**Authors:** Shin Woo Kang, Dong-Hyun Baek, Byeong-Kwon Ju, Young Wook Park

**Affiliations:** 1 Display and Nanosystem Laboratory, School of Electrical Engineering, Korea University, 145, Anam-ro, Seongbuk-gu, Seoul, 02841 Republic of Korea; 2 Nano and Organic-Electronics Laboratory, Department of Display and Semiconductor Engineering, Sun Moon University, Asan, Chungcheongnam-do 31460 Republic of Korea; 3 Center for Next Generation Semiconductor Technology, Department of Display and Semiconductor Engineering, Sun Moon University, Asan, Chungcheongnam-do 31460 Republic of Korea

**Keywords:** Electrical and electronic engineering, Electronics, photonics and device physics, Organic LEDs

## Abstract

In this study, we report highly efficient green phosphorescent organic light-emitting diodes (OLEDs) with ultra-thin emission layers (EMLs). We use tris[2-phenylpyridinato-C2,N]iridium(III) (Ir(ppy)_3_), a green phosphorescent dopant, for creating the OLEDs. Under systematic analysis, the peak external quantum efficiency (EQE) of an optimized device based on the ultra-thin EML structure is found to be approximately 24%. This result is highest EQE among ultra-thin EML OLEDs and comparable to the highest efficiency achieved by OLEDs using Ir(ppy)_3_ that are fabricated via conventional doping methods. Moreover, this result shows that OLEDs with ultra-thin EML structures can achieve ultra-high efficiency.

## Introduction

Research has been progressing on organic light-emitting diodes (OLEDs), ever since they were first developed in 1987 by Tang et al*.*^1^. From then, to the present, OLEDs have attracted remarkable amounts of attention and undergone much development, because of several advantages such as their lightweight, thin, flexible, and stretchable structures, and variable form factors^2–5^. Owing to these advantages, OLEDs are currently attracting much attention in the television, monitor, mobile, and other display markets. However, certain problems remain to be solved. For instance, the devices exhibit problems in terms of their efficiency, lifetime, etc.; their fabrication involves problems related to the long processing time, complicated processes, requirement of high-level vacuum, etc.^6,7^. Much research is being conducted to solve these problems. To increase the efficiency of the OLEDs, the development of organic materials, optimization of the device structure, and improvement of the outcoupling efficiency are necessary. The development of organic materials began with fluorescent materials and proceeded to phosphorescent and thermally activated delayed fluorescent (TADF) materials^8,9^. The maximum achievable internal quantum efficiency (IQE) was 25% for fluorescent devices emitting only a singlet exciton state^10,11^. Phosphorescent devices and TADF devices that utilized triplets for light emission through intersystem crossing (ISC) or reverse intersystem crossing (RISC) could achieve IQEs up to 100%^4,6,10–12^. The triplet excitons of phosphorescent devices had high probabilities of dissociation by meeting each other, because of the long lifetime. The high density of triplet excitons caused triplet–triplet annihilation (TTA) and triplet–polaron quenching (TPQ)^13–16^. In order to prevent TTA and achieve high efficiency, an appropriate doping concentration is required. In a doping method conventionally used in low-molecular OLEDs, a host and dopant were simultaneously deposited on an emission layer (EML) in a specific ratio, which was determined by the weight percent. In other words, the deposition rates of the host and dopant were determined by the doping ratio, which was a difficult process requiring very precise control of the deposition rate and deposition thickness monitoring. Because of this difficulty with regard to doping, research has been conducted to fabricate OLEDs without the use of conventional doping methods^17–20^. Although Xu et al*.* reported 38% EQE with ultra-thin EML, however, it was used tandem structure. Theoretically, the tandem structure is formed by several single units, the EQE and current efficiency of the tandem device increase by X times with the number of single units^20^. Therefore, it should not merely accept the face value.

We fabricated OLEDs with ultra-thin EML structures using tris[2-phenylpyridinato-C2,N]iridium(III) (Ir(ppy)_3_). The fabricated devices were optimized via precise control over the optical path length (OPL) and charge balance of the device structures, without the usage of any light extraction technology. The resulting external quantum efficiency (EQE) of 23.8% showed that OLEDs with ultra-thin EMLs could achieve comparably higher efficiencies than the OLEDs with conventionally doped EMLs. The demonstrated EQE (23.8%) was the highest among that of the devices that did not use tandem structure, novel hosts or transporting layers; it was also comparable to that of the device with the highest efficiency.

## Results

**Figure 1:**
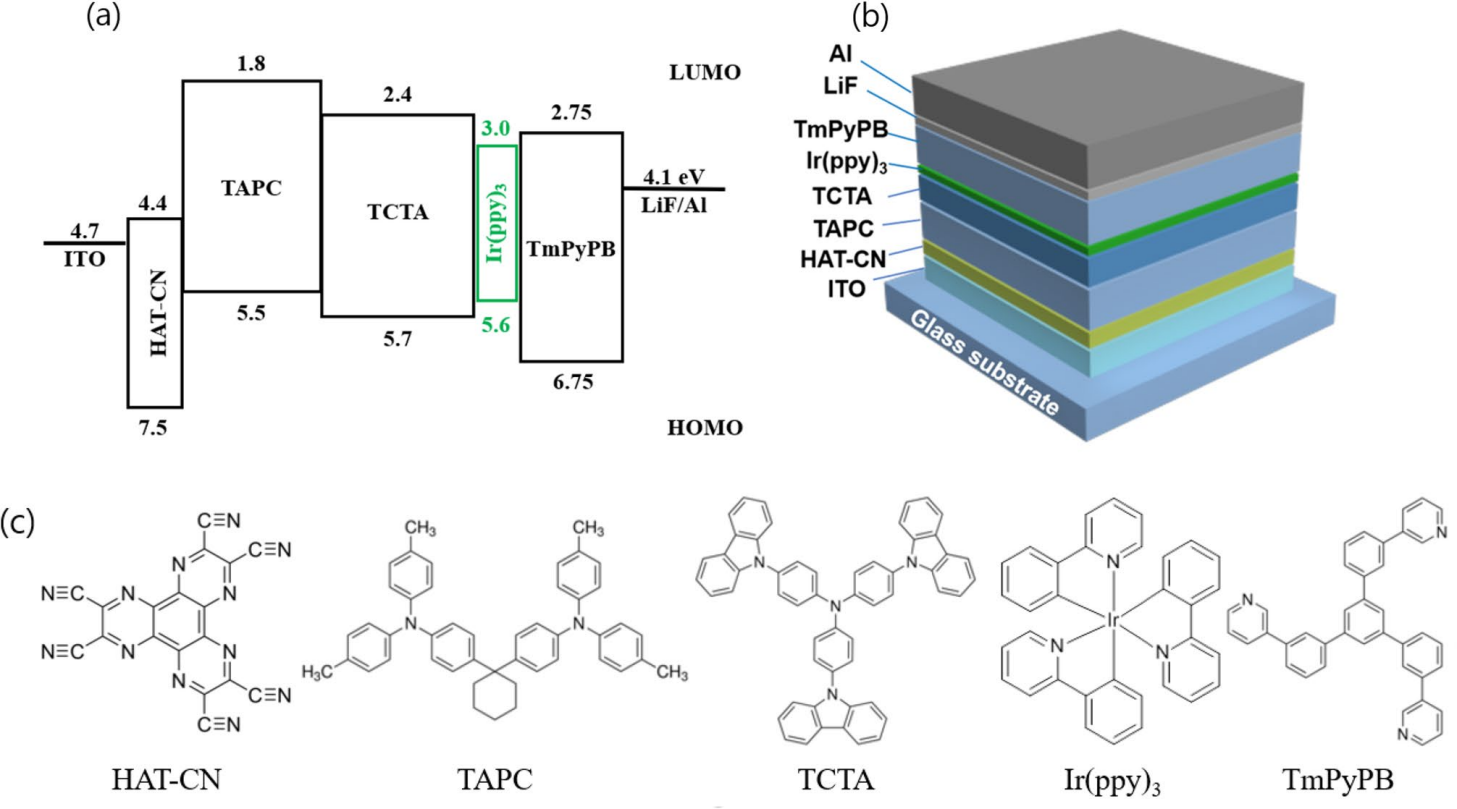
(**a**) Energy level diagram of OLED device with ultra-thin EML structure. (**b**) Fabricated OLED device structure. (**c**) Molecular structures of the organic materials used in the device.

In this study, OLEDs with ultra-thin EMLs were fabricated. The ultra-thin EML structure referred to a structure in which a dopant was inserted into the EML position—for example, between a the hole transport layer (HTL) and electron transport layer (ETL), with very small thickness in the order of several nanometres or less, without the use of a conventional doping method. Figure 1a shows the energy level diagram of the fabricated OLEDs with ultra-thin EML structures, and Fig. 1b shows a schematic of the fabricated OLEDs. Figure 1c shows the molecular structures of the organic materials used in this device.

**Figure 2:**
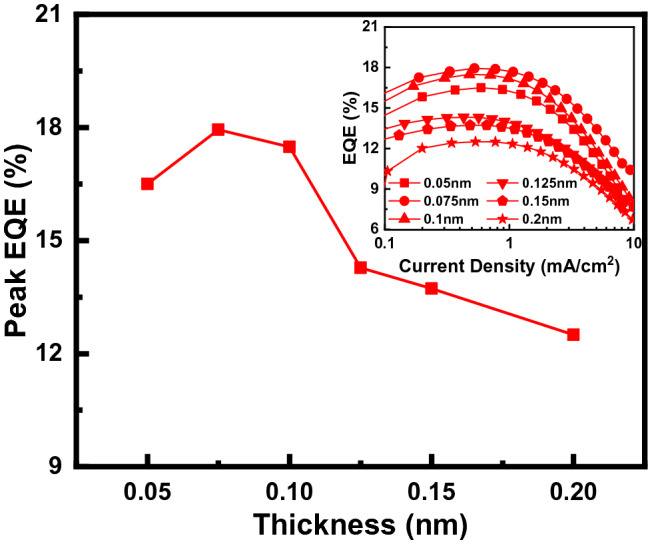
Peak EQE versus thickness of Ir(ppy)_3_ from 0.05 to 0.2 nm. The inset shows the EQE characteristics of the fabricated OLEDs, as a function of current density.

First, OLEDs with the structure dipyrazino[2,3-f:2′,3′-h]quinoxaline-2,3,6,7,10,11-hexacarbonitrile (HAT-CN) 0.5 nm/di-[4-(*N*,*N*-di-p-tolyl-amino)-phenyl]cyclohexane (TAPC) 85 nm/4,4′,4″-Tris(carbazole-9-yl)triphenylamine (TCTA) 30 nm/Tris[2-phenylpyridinato-C2,N]iridium(III) (Ir(ppy)_3_) X nm/1,3,5-Tri[(3-pyridyl)-phen-3-yl]benzene (TmPyPB) 75 nm/lithium fluoride (LiF) 1 nm/aluminum (Al) were fabricated to determine the optimum thin-EML thickness by balancing the energy transfer and trapping emissions while minimizing the TTA. The dopant thickness of X nm was adjusted from 0.05 to 0.2 nm to determine the optimum dopant thickness at which the energy transfer from the adjacent layer and direct light emission by trapping of dopants occurred most efficiently. Figure 2 shows the peak EQE, according to the dopant thickness of the fabricated OLEDs; the inset shows the current–density–EQE characteristics according to the dopant thickness.

We can observe that the efficiency was the highest at the thickness of 0.075 nm. Between 0.05 and 0.10 nm, the peak EQE exhibited peaks, but reduced greatly at larger thicknesses. This suggests that thicknesses greater than 0.1 nm can result in exciton quenching such as TTA^15,16^.

The Ir(ppy)_3_ ultra-thin EML OLEDs were fabricated considering the optimum thickness of 0.075 nm, and were then optimized further. Multiple sets of devices with different thickness of the ultra-thin layer were compared while controlling the thicknesses of the other layers [including hole injection layer (HIL), HTL, ETL, and electron injection layer (EIL)] simultaneously. Among all such devices, the device with the 0.075-nm thick ultra-thin EML showed the highest efficiency, within the boundary of first-order constructive interference. This suggested that the thickness of 0.075 nm was finely optimized for high-efficiency ultra-thin Ir(ppy)_3_ EML OLEDs.

To analyse the electroluminescence (EL) characteristics of OLEDs with ultra-thin EML in more depth, we compared the ultra-thin EML method and conventional doping method of OLEDs. In the two doping methods, the dopant molecules have different molecular distribution. The dopants of ultra-thin EML are distributed in a two-dimensional form (in-planar). However, the hosts and the dopants of conventional doping method are mixed in three-dimensional form. Due to this difference, direct comparison is difficult to conduct. Therefore, the ultra-thin EML method and the conventional doping method are indirectly compared and analysed using the intermolecular distance of dopant molecules, which is an important variable in the host-dopant energy transfer process. The average intermolecular distance of dopants was calculated by the assuming that the ultra-thin EML method and conventional doping method have a uniform molecular distribution in two and three dimensions, respectively [Supporting Information-1].

**Figure 3:**
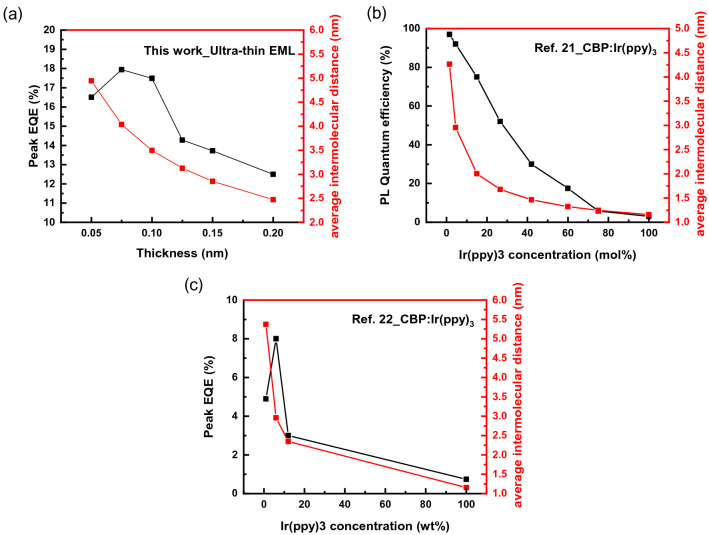
The relation between quantum efficiency and average dopant intermolecular distance according to the thickness of ultra-thin EML and doping concentration (**a**) this work, (**b**) reference ^21^, and (**c**) reference ^22^.

To compare the ultra-thin EML method and the conventional doping method, photoluminescence (PL) and EL quantum efficiency data were referred from in researches result of the CBP:Ir(ppy)_3_ doping structure in the form of a solid-state thin film, and a widely used doping structured phosphorescent OLEDs, respectively^21,22^. Figure 3 shows the relationship between the quantum efficiencies and the average intermolecular distance of dopant according to the dopant thickness in the ultra-thin EML method (this work) and the doping concentration in the conventional doping method (from references^21,22^). As in Fig. 3a, the thickness of 0.075 nm ultra-thin EML, which showed the highest efficiency, was interpreted with average dopant intermolecular distance of 4.0 nm. As we mentioned with Fig. 2, the shorter intermolecular distance of 3.5 nm results the increased TTA and decreased efficiency (0.1 nm ultra-thin EML). These characteristics can be confirmed by the results of other researches (Fig. 3b,c). The maximum PL quantum efficiency of doped thin solid film was showed at the doping concentration of 1.5 mol% and the average dopant intermolecular distance of ~ 4.3 nm. While maximum EL quantum efficiency of phosphorescent OLEDs was showed at the doping concentration of 6 wt% and the average dopant intermolecular distance of ~ 3 nm. And they were decreased with the increased doping ratio and average dopant intermolecular distance. As a summary, the PL quantum efficiency of the thin film and the EQE of the phosphorescent OLEDs showed the high-maximum efficiency with an average dopant intermolecular distance of 3 nm or more, while the efficiency decreased rapidly with the average dopant intermolecular distance of below 3 nm. That is, as the average dopant intermolecular distance decreases, the density of the triplet increases. The increase of the triplet density causes TTA, which in turn leads to decrease efficiency. And these results shows good consistency with TTA theory of high exciton quenching with below 3 nm as in literatures^16^.

**Figure 4:**
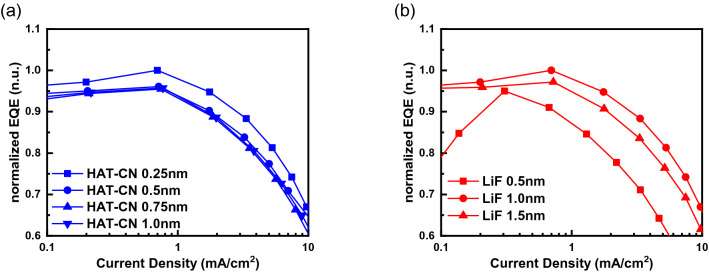
Normalized EQE characteristics as a function of current density: (**a**) HIL (HAT-CN) thickness changed, (**b**) EIL (LiF) thickness changed.

To maximize the efficiency, the injection layer was controlled carefully for improved carrier balance. The initial thickness of the HTL and ETL were calculated and fixed at 80 and 74 nm, respectively, which were calculated to have constructive internal interference OPLs [Supporting Information 2]. Figure 4 shows the normalized (by maximum peak efficiency) EQE characteristics, as a function of the current density. Figures 4a,b show the EL characteristics depending on the thickness of the injection layer, HAT-CN (0.25–1.0 nm) and LiF (0.5–1.5 nm), respectively.

A clear dependence on the injection layer thickness was observed. The 0.25-nm thick HAT-CN and 1.0-nm thick LiF showed the highest efficiencies. Between the HIL and EIL, the EIL showed a much larger dependence, which meant that this device structure was hole dominant and carrier unbalanced. This result also suggested that much enhancement could be achieved by optimizing the HTL and ETL thicknesses or by using a high-mobility ETL^23,25^.

**Figure 5:**
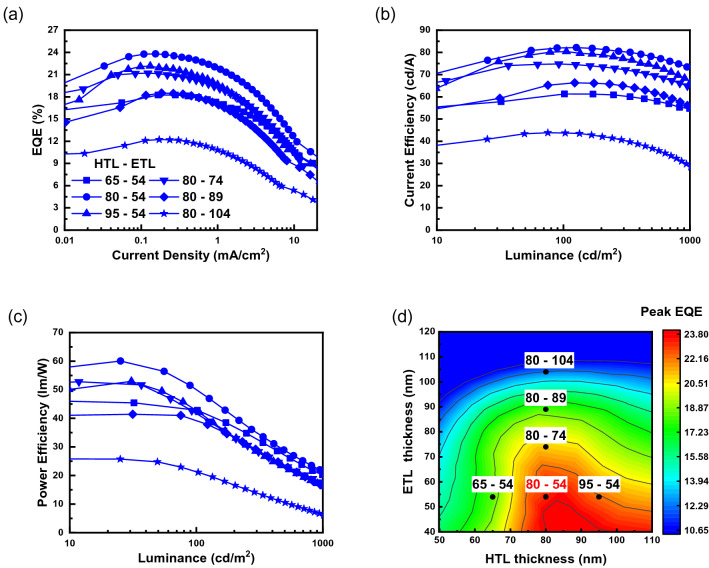
EL characteristics of six representative devices: (**a**) current density–EQE (J*–*EQE) characteristics, (**b**) current efficiency–luminance (CE–L) characteristics, and (**c**) power efficiency–luminance (PE–L) characteristics. (**d**) EQE contour plot of all the fabricated OLEDs with various HTL and ETL thickness (the dots are data of the six-representative device).

However, modifying the carrier balance by controlling the thickness of the transport layer could have a negative effect on the internal interference matching of the OPL. Therefore, we investigated the correlation between the optical efficiency (constructive interference) and carrier balance. The thicknesses of the dopant, HIL, and EIL were fixed at 0.075, 0.25, and 1.0 nm, respectively. The thicknesses of the HTL and ETL were step-wise varied from the calculated values; the HTL thickness was varied from 65 to 95 nm, and the ETL thickness from 54 to 104 nm. Figures 5a–c show the EL characteristics of six representative devices (HTL–ETL: 65–54, 80–54, 95–54, 80–74, 80–89, 80–104). Figure 5d shows a summary of the peak EQE data of all OLEDs. The detailed EL characteristics of the fabricated OLEDs are presented in the supporting information [Supporting Information-3].

In Fig. 5, among the HTL-controlled devices of different thicknesses, the 80-nm thick HTL showed the highest efficiency, as determined in Supporting Information—3. However, the effect of thickness of the ETL was different. On comparing the devices of 80–54, 80–74, 80–89, and 80–104, the EL efficiencies decreased following an increase in ETL thickness. This is verified by Fig. 5d, which presents the EQEs of all fabricated devices. The decrease in efficiency caused by an increase in ETL thickness could be due to the carrier imbalance. In this structure, this signifies that the mobility of the ETL is much lower than that of the HTL. In addition, as presented in Supporting Information-3, the device is hole-dominant. Therefore, the ETL must be thinner than the HTL. This result suggests that the carrier balance is dominant in this device structure, and that the optical efficiency affected by the internal interference is less effective in here. The peak EQE dependence on the HTL and ETL thickness is summarized and presented in Table 1.

**Table 1:** Summary of peak EQE for different ETL and HTL thickness.

	ETL thickness			
	54 nm (%)	74 nm (%)*	89 nm (%)	104 nm (%)
**HTL thickness**				
65 nm	18.2^b^	18.8	16.2	10.7
80 nm^a^	23.8^b^	21.2^b^	18.5^b^	12.2^b^
95 nm	22.2^b^	19.7	17.4	12.2

^a^Reference thickness calculated using refractive index for constructive interference.

^b^Devices presented in Fig. 5a–c.

The optical and electrical optimizations of the ultra-thin EML OLEDs were conducted by adjusting the thickness of the dopant, ETL, and HTL. The maximum EQE of the fabricated OLEDs with the ultra-thin Ir(ppy)_3_ EML structure was 23.8%, and the device was fabricated without employing a doping process. However, as shown in the graph in Fig. 5a, an EQE roll-off was observed at high current densities. At a current density of 10 mA/cm^2^, the EQE roll-off was ~ 50% of the peak EQE. Nevertheless, the proposed device achieved 23.8% of maximum EQE, which was the highest EQE, when compared to that of the previously reported ultra-thin OLEDs^17,19^. The efficiency was relatively higher than that of the conventional doping methods that did not use a novel host or transporting layer^24^ it was also comparable to that of the highest efficiency device^25^. Table 2 shows a summary of the peak EQE characteristics of the Ir(ppy)_3_ devices with highest efficiency.

**Table 2:** Summary of peak EQE in this study and in previous works.

	Peak EQE (%)	Doping method	Dopant	Note
This work	23.8	Undoped (ultra-thin EML)	Ir(ppy)_3_	0.075 nm thickness of dopant TCTA/TmPyPB interface
^17^	20.9	Undoped (ultra-thin EML)	Ir(ppy)_2_acac	0.1 nm thickness of dopant TCTA/TmPyPB interface
^19^	21.1	Undoped (ultra-thin EML)	Ir(ppy)_2_acac	0.3 nm thickness of dopant TCTA/TmPyPB interface
^24^	21.2	Conventional	Ir(ppy)_3_	Using novel host Ir(ppy)_3_ 6 wt% DCzBPI
^25^	25.5	Conventional	Ir(ppy)_3_	Heavy doping and high mobility ETL ETL: B4PyPPM Ir(ppy)_3_ 17 wt% doped CBP

The OLED structure used in this study did not satisfy the optical path and charge balance requirements, simultaneously, and the EQE roll-off phenomenon occurred; nevertheless, the results demonstrated the highest EQE, when compared to that of other reports^17,19,24,25^.

The strategy for inserting ultra-thin dopants into high-efficiency OLEDs is simple, and is summarized below.

1. The thickness of ultra-thin dopant with highest efficiency is determined.
2. Using a simple optically optimized structure (transport layer thickness, HTL–ETL), the thickness of the HIL–EIL that can provide the highest efficiency is determined, and the refractive-index difference and mobility are disregarded.
3. The HTL–ETL thickness is finely optimized, considering the refractive-index difference and mobility.

Therefore, using alternative high-mobility ETL materials, low-driving-voltage materials, etc., and applying the wide-emission-zone method utilizing multiple insertions of ultra-thin EMLs can enable the requirements of optical path and charge balance to be satisfied, simultaneously, producing OLEDs with high efficiency and low efficiency roll-off.

## Conclusion

In this study, highly efficient phosphorescent OLEDs were fabricated by inserting Ir(ppy)_3_ dopants in the form of ultra-thin films, without using conventional doping methods. In order to maximize the IQE of OLEDs with ultra-thin EML structures, the dopant thickness was controlled precisely, and the thicknesses of the injection and charge-transport layers were changed systematically for analysing and characterizing the optical efficiency induced by internal interference and charge-carrier balance. As a result, the proposed device exhibited one of the highest levels of EQE (23.8%), among the observed research results of phosphorescent OLEDs using Ir(ppy)_3_ that did not employ any light-extraction technology or other novel layer materials. This exemplary result could be owed to the complex contribution of efficient energy transfer, minimization of exciton dissipation, and optimization of optical internal interference structures and charge balance. This suggests that the OLED devices with ultra-thin EML structures can achieve ultra-high efficiency, comparable to that of the conventional doping technologies. By combining the proposed technology with other technologies, further research and enhancement can be expected, including reduced efficiency roll-off and high power efficiency.

## Methods

The OLEDs were fabricated on a commercially purchased indium tin oxide (ITO)-coated STN-LCD glass substrate. The substrates were pre-cleaned through ultrasonication using acetone, methanol, and DI water, in that order. Before thermal evaporation was conducted, the surface of ITO was treated using UV ozone and O_2_ plasma, in sequence. The structure of the OLEDs is described as follows. ITO 185 nm in thickness was used as the anode, HAT-CN was used as the HIL, and TAPC and TCTA were used as the HTL. Ir(ppy)_3_ was used as the ultra-thin EML, TmPyPB was used as the ETL, LiF was used as the EIL, and Al was used as the cathode. All the organic materials were purchased from Sigma-Aldrich and Lumtec Corp. The vacuum level of the process chamber was maintained at ~ 3 × 10^–7^ Torr. During the evaporation of all materials, the substrates were rotated at a constant speed of 12 rpm. The organic materials evaporated at a rate of ~ 1 Å/s, and the emitting dopant evaporated at a rate of ~ 0.01 Å/s. LiF evaporated at a rate of ~ 0.1 Å/s, and Al evaporated at a rate of ~ 4 Å/s. The fabricated devices were stored in a glove box, in an Ar atmosphere containing less than 1 ppm of H_2_O. A 6-MHz gold-coated quartz crystal microbalance (QCM) and a thin-film deposition controller with a PCI Express interface (IQM-233, INFICON) were used to detect the thickness of the film during the high-vacuum thermal deposition of each thin film. The thickness of the organic materials was measured under an over 98% lifetime of QCM. For precise control of the ultra-thin films, the thickness of each film was calibrated with a thick film of thickness over 500 nm, using a field-emission scanning electron microscope (JSM-6700F, JEOL Co. Ltd) aided by a surface profilometer (Alpha-Step 500, KLA-Tencor). The EL characteristics of the fabricated OLEDs were measured using a spectroradiometer (CS-1000, Konica Minolta Co., Ltd.) and source meter (Keithley-2400, Tektronix) in a dark box at 25 degrees Celsius (°C) and air atmosphere. The EL efficiency was calculated under the assumption that the OLED was a Lambertian light source.

## Supplementary Information

Supplementary Information
