# Supplementary material for: Green phosphorescent organic light-emitting diode exhibiting highest external quantum efficiency with ultra-thin undoped emission layer
Source: Sci Rep. 2021 Apr 19;11:8436. doi: 10.1038/s41598-021-86333-9 (PMC8055988; doi:10.1038/s41598-021-86333-9)
Supplement: Supplementary file 1 — Supplementary Information [file 41598_2021_86333_MOESM1_ESM.docx]

**(Supporting Information)**

Journal: *Scientific Reports*

Green Phosphorescent Organic Light-Emitting Diode Exhibiting Highest External Quantum Efficiency with Ultra-thin Undoped Emission Layer

Shin Woo Kang^1,2^, Dong-Hyun Baek^3^, Byeong-Kwon Ju^1,*^ and Young Wook Park ^2,* *^

^1^Display and Nanosystem Laboratory, Department of Electrical Engineering, Korea University, 145, Anam-ro, Seongbuk-gu, Seoul 02841, Republic of Korea

^2^Nano and Organic-Electronics Laboratory, Department of Display and Semiconductor Engineering, Sun Moon University, Asan, Chungcheongnam-do 31460, Republic of Korea

^3^Center for Next Generation Semiconductor Technology, Department of Display and Semiconductor Engineering, Sun Moon University, Asan, Chungcheongnam-do 31460, Republic of Korea

[*] Prof. Byeong-Kwon Ju

Laboratory Homepage: http://diana.korea.ac.kr

E-mail: [bkju@korea.ac.kr](mailto:bkju@korea.ac.kr)

Phone No.: +82-2-3290-3237, Fax. No.: +82-2-3290-3791

[**] Prof. Young Wook Park

E-mail: [zerook@sunmoon.ac.kr](mailto:zerook@sunmoon.ac.kr)

Phone No.: +82-41-530-2365, Fax. No.: +82-41-530-2365

1. **Calculation of the dopant intermolecular distance**

The dopants of ultra-thin EML structure in this work, are distributed in a two-dimensional form(in-planar). However, the hosts and the dopants of the conventional doping method are mixed in three-dimensional form. Due to this difference, direct comparison is difficult to conduct. Therefore, to indirectly compare the ultra-thin EML method and the conventional doping method, the intermolecular distance of dopant molecules, which is an important variable in the host-dopant energy transfer process, was calculated. In the case of the ultra-thin EML method, it is assumed that the dopant thin film is composed of a monolayer of molecules, and the dopant molecules are evenly distributed in the centre of the two-dimensional lattice. And the conventional doping method, it is assumed that the distribution of dopant molecules is uniformly distributed around the dopant in the three-dimensional, and the dopant molecules are located the centre of the cubic. The average intermolecular distance of dopants was calculated by assuming that each doping method has a uniform molecular distribution in two and three dimensions.


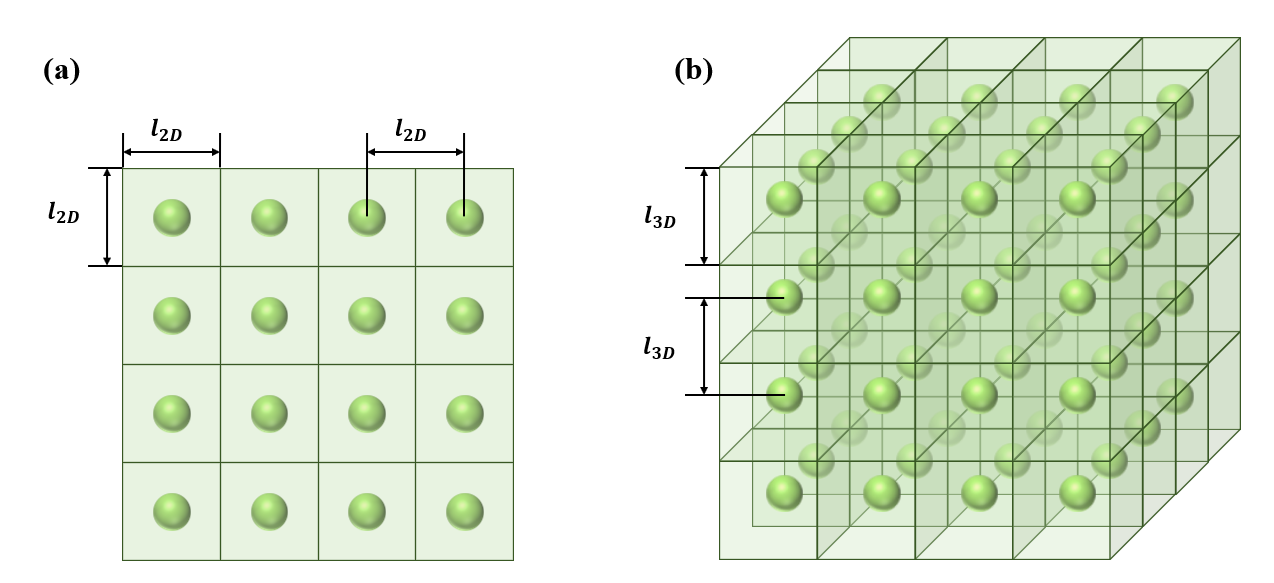


**Supplementary Figure S1. Schematic of assumption-based dopant molecular arrangement in (a) 2-dimensional ultra-thin EML structure, and (b) 3-dimensional conventional doping structure.**

Fig. S1 shows the simplified dopant molecular distribution in 2D and 3D. The average intermolecular distance of dopants which is uniformly distributed in 2D and 3D was calculated by the below equations. Divide the number of deposited dopant molecules by the total deposition area of volume to obtain the area and volume occupied by one dopant molecule and calculated the area and volume of the occupied dopant lattice uniformly distributed in 2D and 3D. Assuming the lattice distance and the average intermolecular distance of dopant are equal, and the average intermolecular distance in 2D and 3D distribution were calculated. $m_{dopant}$is the deposited mass of dopants, $V_{tot}$ is the volume of ultra-thin EML (dopant thin film) or doped EML,$d_{dopant}, n_{dopant}$, $M_{dopant}$ are the density of dopant, number of deposited dopant molecules, and molecular weight of dopant(the molecular weight of Ir(ppy)_3_ = 654.78 g/mol), respectively. $N$ is the Avogadro number(6.02214076 × 10^23^ mol^−1^), $A_{tot}$ is the total deposited area, $A_{dopant}$,$l_{2D}$ are the occupied area of dopant molecule in 2D distribution and intermolecular distance of dopant in 2D distribution respectively, $V_{dopant}$, $l_{3D}$ are the occupied volume of dopant molecule in 3D distribution, and intermolecular distance of dopant in 3D distribution respectively.

$m_{dopant} =V_{tot}\times d_{dopant}$ (1)

$n_{dopant}=\frac{m_{dopant}}{M_{dopant}}\times N$ (2)

$A_{dopant}=\frac{A_{tot}}{n_{dopant}}$ (3)

$l_{2D}=\sqrt{A_{dopant}}$ (4)

$V_{dopant}=\frac{V_{tot}}{n_{dopant}}$ (5)

$l_{3D}=\sqrt[3]{V_{dopant}}$ (6)

**Supplementary Figure S2.** The relation between quantum efficiency and average dopant intermolecular distance according to the thickness of ultra-thin EML and doping concentration (a)this work, (b)reference 1, (c)reference 2. The dash-dot line shows the critical distance at 70% of maximum efficiency.

**Supplementary Table S1.** Summary of average dopant intermolecular distance and efficiency characteristics of doping method in this work and references.

|  | Experimental (EL) | Ref. 1 (PL) | Ref. 2 (EL) |
| --- | --- | --- | --- |
| Doping method | Undoped  *Ultra-thin EML | Conventional  *EML Thin Film | Conventional  *PHOLEDs |
| Host:dopant | Thin film in TcTa/TmPyPB interlayer | CPB:Ir(ppy)­_3_ | CPB:Ir(ppy)­_3_ |
| Peak efficiency at Ir(ppy)_3_ concentration/thickness_­_ | 0.075 nm | 1.5 mol% | 6 wt% |
| Calculated intermolecular distance | 4.0 nm | 4.3 nm | 3 nm |
| Critical distance  (at 70% of maximum efficiency) | 2.5 nm | 1.9 nm | 2.6 nm |

To compare the ultra-thin EML method and the conventional doping method, photoluminescence (PL) and electroluminescence (EL) quantum efficiency data were referred from in researches result of the CBP:Ir(ppy)­_3_ doping structure in the form of a solid-state thin film, and a widely used doping structure. [1-2] Fig. S2 shows the relationship between the quantum efficiencies and the average intermolecular distance of dopant according to the dopant thickness in the ultra-thin EML method(this work) and the doping concentration in the conventional doping method(from references [1-2]). A summary of characteristics such as doping concentration, the thickness of dopant thin film, and intermolecular distance of dopant when maximum efficiency is exhibited for each doping method is shown in Table. S1. As in Fig. S2 (a), the thickness of 0.075 nm ultra-thin EML, which showed the highest efficiency, was interpreted with the average dopant intermolecular distance of 4.0 nm. And in Figs (b) and (c), The maximum PL quantum efficiency of the doped thin solid film was shown at the doping concentration of 1.5 mol% and the average dopant intermolecular distance ~ 4.3 nm. While maximum EL quantum efficiency of PHOLEDs was showed at the doping concentration of 6wt% and the average dopant intermolecular distance of ~3 nm. PL quantum efficiency and EQE characteristic are difficult to directly compare by using the intermolecular distance of dopant because of various factors such as different thin film composition, energy transfer process in the host-dopant system, and carrier balance. However, it was confirmed that the EQE characteristics of the OLEDs with ultra-thin EML structure and the OLEDs with conventional doping structure showed a similar tendency according to the intermolecular distance of dopant. As in Fig. S2 and Table. S1, In the ultra-thin EML method and conventional doping method, the average dopant intermolecular distances at 70% of maximum EQE were 2.5 nm and 2.6 nm, respectively, showing very similar.

1. **Calculation of optical path length – thickness of HTL and ETL**

Using the centre wavelength of the Ir(ppy)_3_ photoluminescence (PL) spectra half maximum, 528 nm, [3] the ETL and HTL thicknesses were calculated to obtain a constructive interference optical path length (OPL).

Supplementary Fig. S3 shows a schematic diagram of the conditions under which the light emitted from the EML reflects off the cathode, causing constructive interference. In equation (7) presented below, *n* represents the refractive index, and *d* is the geometric length of the path followed by the light inside of OLED devices, such as the thicknesses of ITO or organic layers. The refractive-index value was taken from the data of Salehi, A et al. [4].


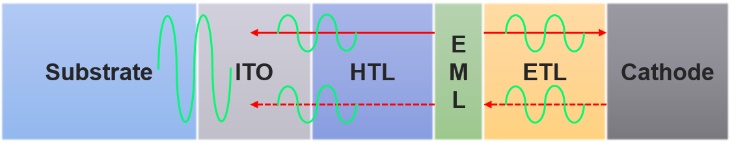


Supplementary Figure S3. Schematic diagram of constructive interference mechanism in the OLED structure.

$OPL=n\times d$ (7)

$\Delta\phi= \frac{2\pi}{\lambda}\left( 2nd \right)-\pi=2\pi m$ (m = 0, 1, 2, 3, …) (8)

$T_{ETL}=\frac{\lambda}{2n}\left( m+\frac{1}{2} \right), (m=0)$ (9)

$T_{tot}=\frac{\lambda}{2n}\left( m+\frac{1}{2} \right), \left( m=2 \right)$ (10)

$T_{HTL}=T_{tot}- T_{ETL}- T_{EML}-T_{ITO}$ (11)

Equation (8) gives the phase change condition for constructive interference. *T*_ETL_ in equation (9) represents the thickness of the ETL, and *T*_tot_ in equation (10) is the sum of the thicknesses of the ITO, HTL, EML, and ETL. In the above schematic and equations, the thickness of the electron injection layer (EIL) and hole injection layer (HIL) were not considered because they were much thinner than the other layers. The thickness of the EML (*T*_EML_) was assumed to be 30 nm. The refractive indexes of the whole organic layers were assumed to be 1.79. [4] The thickness of the ITO (*T_ITO_*) was 185 nm and the centre wavelength of the Ir(ppy)_3_ PL spectra half maximum was 528 nm. [3] The thicknesses of the ETL and HTL were calculated using the above equations. The thickness of the ETL and HTL were chosen to be 74 nm (*m* = 0) and 80 nm (*m* = 2), as calculated in Tables S2 to S4. The first, harmonic order and the lowest thickness was selected for achieving low driving voltage and high efficiency.

Supplementary Table S2. Calculated thicknesses of ETL.

| Colour | Wavelength | *m*= 0 | *m* = 1 | *m* = 2 | *m* = 3 |
| --- | --- | --- | --- | --- | --- |
| Red | 620 nm | 87 | 260 | 433 | 606 |
| Green | 535 nm | 75 | 224 | 374 | 523 |
| Blue | 420 nm | 59 | 176 | 293 | 411 |
| Green-Ir(ppy)_3_ | 528 nm | 74 | 221 | 369 | 516 |

Supplementary Table S3. Calculated thicknesses of all layers.

| Colour | Wavelength | *m* = 0 | *m* = 1 | *m* = 2 | *m* = 3 |
| --- | --- | --- | --- | --- | --- |
| Red | 620 nm | 87 | 260 | 433 | 606 |
| Green | 535 nm | 75 | 224 | 374 | 523 |
| Blue | 420 nm | 59 | 176 | 293 | 411 |
| Green-Ir(ppy)_3_ | 528 nm | 74 | 221 | 369 | 516 |

Supplementary Table S4. Calculated thicknesses of HTL.

| Colour | Wavelength | *m* = 0 | *m* = 1 | *m* = 2 | *m* = 3 |
| --- | --- | --- | --- | --- | --- |
| Red | 620 nm | −202 | −65 | 114 | 317 |
| Green | 535 nm | −214 | −113 | 85 | 234 |
| Blue | 420 nm | −230 | −68 | 4 | 122 |
| Green-Ir(ppy)_3_ | 528 nm | −215 | −68 | 80 | 227 |

1. **EL characteristics of ultra-thin OLEDs for various HTL and ETL thicknesses**

Supplementary Figure S4. EL characteristics of fabricated OLEDs: (a) Current density–voltage–luminance (*J–V–L*) characteristics, (b) external quantum efficiency–current density (EQE–*J*) characteristics, (c) current efficiency–luminance (CE–L) characteristics, and (d) power efficiency–luminance (PE–L) characteristics.

Supplementary Fig. S4 shows the EL characteristics of the fabricated OLEDs. Supplementary Fig. S4 (a) shows a clear increase in the driving voltage as the thickness of the transport layer increases. However, the dependence on the thickness is different for the HTL and ETL. The driving voltage increase of the ETL is larger than that of the HTL, when the thickness of the HTL is lesser. The details of the driving voltage are presented in Supplementary Table S5.


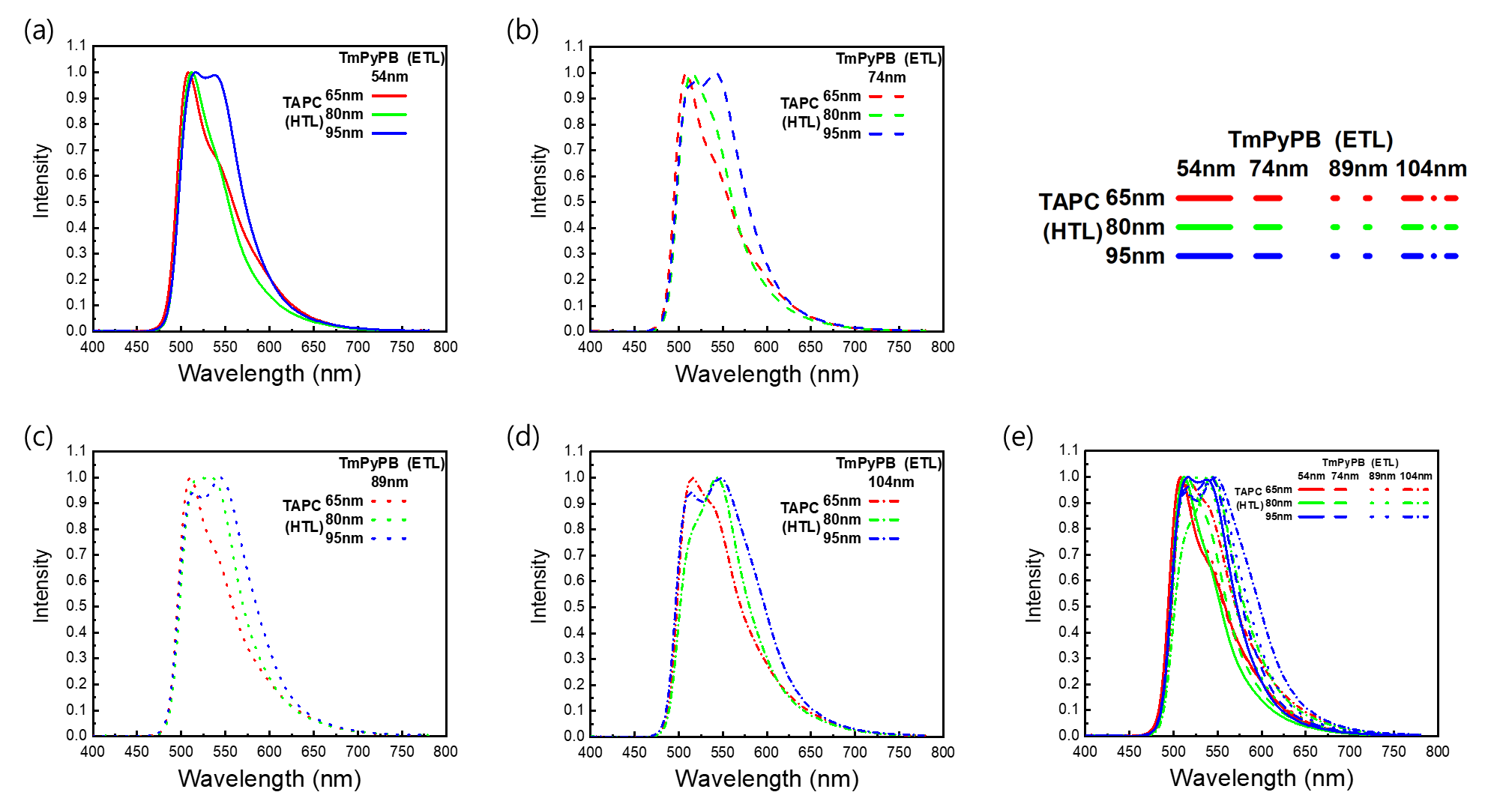


Supplementary Figure S5. EL spectra data of the fabricated OLEDs devices with different HTL and ETL thickness combinations

Supplementary Table S5. Summary of driving voltage at 10 mA/cm^2^ and driving voltage increase

| Device Configuration | *V* at 10 mA/cm^2^ | | | | |
| --- | --- | --- | --- | --- | --- |
|  | ETL 54 | ETL 74 | ETL 89 | ETL 104 | △*V*  (ETL 104-ETL 54) |
| HTL 65 | 13.9 | 14.7 | 15.9 | 17.9 | 4 |
| HTL 80 | 17.6 | 18.8 | 17.5 | 19.1 | 1.5 |
| HTL 95 | 20.9 | 21.1 | 21.4 | 22.5 | 1.6 |
| △*V*  (HTL 95-HTL 65) | 7 | 6.4 | 5.5 | 4.6 |  |

Supplementary Figs S4 (b-e) show the efficiency characteristics of the OLEDs, in terms of the EQE, current efficiency, and power efficiency. The ETL and HTL show different tendencies for EL efficiency. When the thickness of the ETL increases, the EL efficiency decreases, whereas, the HTL shows the peak EL efficiency at 80 nm thickness. This suggests that the electronic carrier balance is not matched, which results in higher efficiency at the least thickness of ETL. The highest efficiency is achieved when the HTL thickness is 80 nm, which is the same as that obtained through the calculation results. Thus, an alternative material with higher electron mobility and lower injection barrier is required for the ETL to satisfy the requirements of both OPL calculation and device EL efficiency.

In summary, the increase in the driving voltage is much higher for an increase in the thickness of the ETL than for the HTL, which suggests that the device structure is hole-dominant. The decrease in efficiency caused by an increase in ETL thickness is due to the carrier imbalance. Despite the carrier imbalance, the demonstrated OLEDs with 80 nm HTL and 54 nm ETL achieve 23.8% peak EQE.

**Supporting Information References**

1. Kawamura, Y. *et al.* 100% phosphorescence quantum efficiency of Ir (III) complexes in organic semiconductor films. *Appl. Phys. Lett.* **86,** 071104 (2005).
2. Baldo, M. A., Lamansky, P. E., Burrows, P. E., Thompson, M. E. & Forrest, S. R. Very high-efficiency green organic light-emitting devices based on electrophosphorescence. *Appl. Phys. Lett.* **75(4)** (1999).
3. Mehata, M. S. *et al.* Spin mixed charge transfer states of iridium complex Ir(ppy)_3_: transient absorption and time-resolved photoluminescence. *RSC Adv*. **5,** 34094 (2015).
4. Salehi, A., Chen, Y., Fu, X., Peng, C. & So, F. Manipulating refractive index in organic light emitting diodes. *ACS Appl. Mater. Interfaces* **10(11),** 9595-9601 (2018).
